# Analysis of clinical characteristics of mesalazine-induced cardiotoxicity

**DOI:** 10.3389/fphar.2022.970597

**Published:** 2022-09-15

**Authors:** Junyu Chen, Tengfei Duan, Weijin Fang, Shikun Liu, Chunjiang Wang

**Affiliations:** Department of Pharmacy, The Third Xiangya Hospital, Central South University, Changsha, China

**Keywords:** mesalazine, cardiotoxicity, inflammatory bowel disease, myocarditis, pericarditis

## Abstract

**Background:** Mesalazine is the first-line inflammatory bowel disease (IBD) treatment. However, it can cause fatal cardiotoxicity. We aimed to analyze the clinical characteristics of mesalazine-induced cardiotoxicity and provide evidence for clinical diagnosis, treatment, and prevention.

**Methods:** We collected Chinese and English literature on mesalazine-induced cardiotoxicity from 1970 to 2021 for retrospective analysis.

**Results:** A total of 52 patients (40 males and 12 females) were included, with a median age of 24.5 years (range 9–62) and a median onset time of 14 days (range 2–2880). Cardiotoxicity manifested as myocarditis, pericarditis, and cardiac pericarditis. The main clinical manifestations are chest pain (82.7%), fever (46.2%), and respiratory symptoms such as dyspnea and cough (40.4%). The levels of troponin T, creatine kinase, C-reactive protein, leukocyte count, erythrocyte sedimentation rate, and other biochemical markers were significantly increased. Cardiac imaging often suggests myocardial infarction, pericardial effusion, myocardial necrosis, and other symptoms of cardiac injury. It is essential to discontinue mesalamine immediately in patients with cardiotoxicity. Although corticosteroids are a standard treatment option, the benefits remain to be determined. Re-challenge of mesalamine should be carefully considered as cardiotoxic symptoms may reoccur.

**Conclusion:** Mesalazine may cause cardiotoxicity in patients with inflammatory bowel disease, which should be comprehensively diagnosed based on clinical manifestations, biochemical indicators, and cardiac function imaging examinations. Mesalazine should be immediately discontinued, and corticosteroids may be an effective treatment for cardiotoxicity.

## 1 Introduction

Inflammatory bowel disease (IBD) is characterized by chronic recurrent gastrointestinal inflammation, including ulcerative colitis and Crohn’s disease. Ulcerative colitis symptoms include diarrhea, proctorrhagia, tenesmus, urgency, and fecal incontinence, depending on the extent and severity of the disease (Magro et al., 2017). The symptoms of Crohn’s disease vary but typically include abdominalgia, weight loss, and chronic diarrhea (Gomollon et al., 2017). 5-aminosalicylate (5-ASA) is the first-line recommended drug for IBD treatment. Other therapeutic drugs include corticosteroids, immunosuppressants, and tumor necrosis factor (TNF) therapies (Bressler et al., 2015).

5-ASA, also termed mesalazine, is often associated with fever, diarrhea, abdominalgia, and hematochezia (Matsumoto and Mashima, 2020). However, cardiotoxicity has been reported as a rare but potentially fatal adverse reaction (Kristensen et al., 1990). At present, mesalazine-related cardiotoxicity is reported primarily as case reports. Its incidence, clinical features, treatment, and prognosis are still unclear. This study aimed to summarize and analyze the clinical characteristics of mesalazine-associated cardiotoxicity. Data were synthesized based on published studies to provide a reference for the rational use of mesalazine in practice.

## 2 Methods

### 2.1 Search strategy

The following databases were searched: China National Knowledge Infrastructure (CNKI), Wanfang Data, Chinese VIP databases, Web of Knowledge, PubMed, Elsevier, and Embase. The search keywords were “salazosulfapyridine” OR “mesalazine” OR “mesalamine” OR “balsalazide” OR “olsalazine” AND “myocarditis” OR “pericarditis” OR “carditis.” The publication languages were restricted to Chinese and English, and the publication period was from 1 January 1970 to 31 December 2021.

### 2.2 Inclusion and exclusion criteria

Inclusion criteria were case reports and analyses published as full text in peer-reviewed journals. Exclusion criteria were reviews, animal studies, mechanism studies, preclinical studies, duplicate reports, and articles with insufficient data.

### 2.3 Data extraction

Two investigators independently selected the articles based on inclusion and exclusion criteria, followed by a panel discussion. The following data were extracted using a self-designed data extraction table: country, sex, age, primary disease, concomitant medication, mesalazine use and dosage, administration route, onset time, clinical manifestations, laboratory examination, imaging examination, treatment, and prognosis.

### 2.4 Literature quality evaluation

The quality of the 51 studies included was evaluated using the case series evaluation scale recommended by the National Institute for Clinical Excellence (NICE). The assessment consists of whether: 1) the cases originated from multiple treatment centers; 2) the research objectives were clearly described; 3) the inclusion and exclusion criteria were clear; 4) the definitions of the reported outcomes were clear; 5) prospective studies were performed; 6) patients were recruited continuously; 7) the main findings were clearly described; 8) the results were stratified analyses. A “yes” or “no” decision was assigned to each item, with 1 or 0 points. The scores were then aggregated.

## 3 Results

### 3.1 Basic information

**FIGURE 1:**
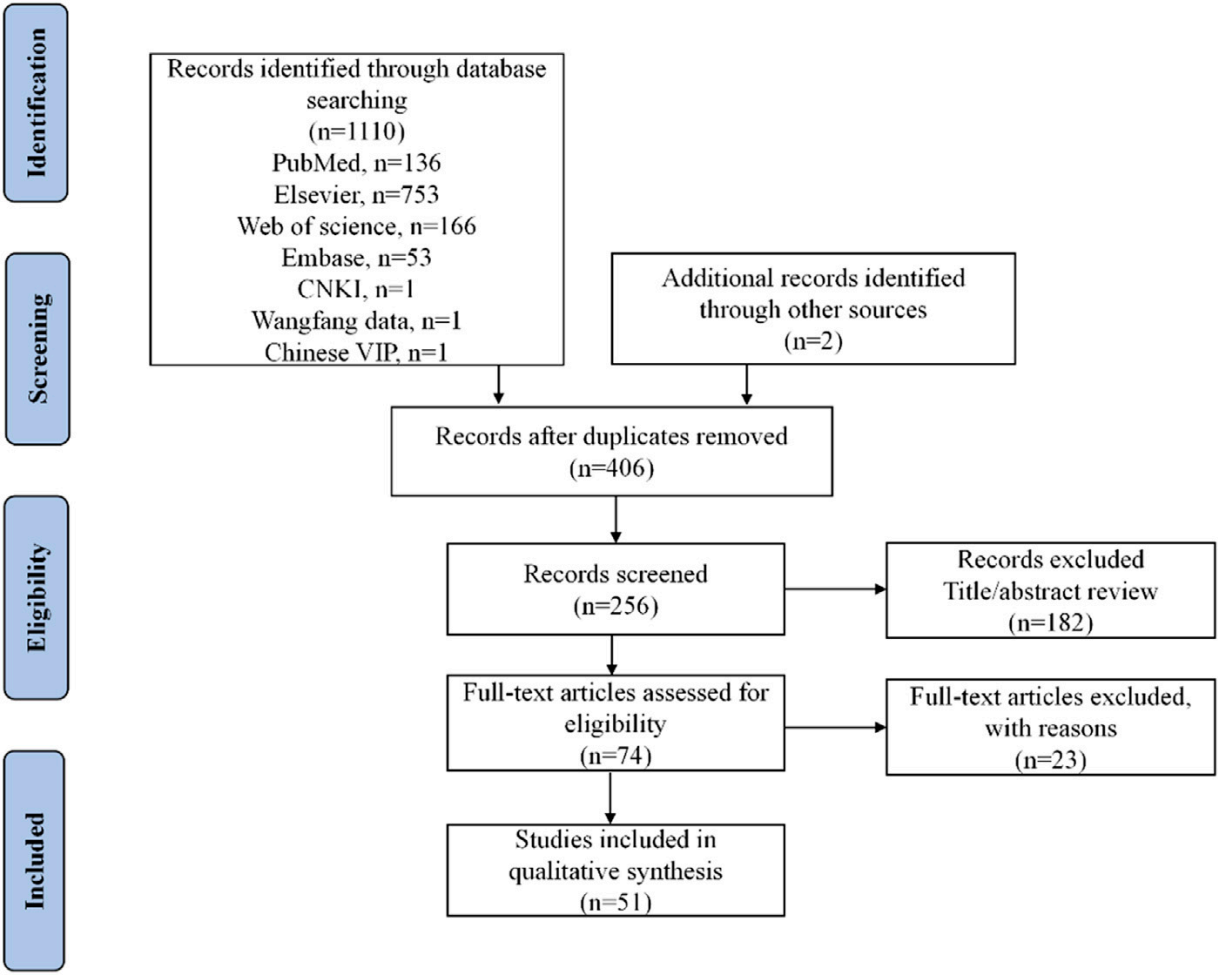
Flow diagram of the selection of studies for inclusion.

After retrieval and screening, 51 studies involving 52 patients (40 men and 12 women) were included, all published in English. The specific methodology for article selection is illustrated in Figure 1. The quality of the 51 articles was evaluated, 50 were rated 3 points, and one rated 2 points. The median age of these patients was 24.5 years (range 9–62), and the median onset time of cardiotoxicity was 14 days (range 2–2880) (Table 1). Thirty-six patients reported the use and dosage of mesalamine in the literature, out of which 6 cases (16.7%) received <2 g/d, 3 (8.3%) received >4 g/d, and the remaining 27 patients (75.0%) received 2–4 g/d. All 36 patients (100.0%) received oral administration, and 5 (13.9%) received rectal administration. Thirty-five cases (67.3%) were indicated for ulcerative colitis (UC), 15 (28.8%) for Crohn’s disease (CD), and 2 (3.9%) for IBD. The underlying diseases of 5 cases (31.2%) were infectious diseases, 5 (31.2%) had blood system disease, 3 (18.8%) had cardiovascular disease, and 3 (18.8%) had skin disease. Twenty-eight cases (53.8%) received concomitant corticosteroids, 6 (11.5%) received antibacterials, and 9 (17.3%) received other drugs for UC, such as azathioprine, sulfasalazine, and others. The other combined medications are shown in Table 1.

**TABLE 1:** General data of 52 patients reported in case series/reports.

Parameter		Value
Age (52)^a^	Years	24.5 (9,62)^b^
Sex (52)^a^	Male	40 (76.9%)
	Female	12 (23.1%)
Region (52)^a^	Europe (United Kingdom, Germany, Portugal, Denmark, Spain, Italy, France)	26 (50.0%)
	Americas (America, Canada)	19 (36.5%)
	Asia (Japan, China, Turkey, Israel)	7 (13.5%)
Onset time (52)^a^	days	14 (22,880)^b^
Use and dosage (36)^a^	Daily dose	
	<2 g	6 (16.7%)
	2g∼4 g	27 (75.0%)
	>4 g	3 (8.3%)
	Usage	
	Oral	36 (100.0%)
	Rectal	5 (13.9%)
Indication (52)^a^	UC	35 (67.3%)
	CD	15 (28.8%)
	IBD	2 (3.9%)
Diseases (16)^a^	Infectious diseases: pancreatitis, arthritis, otitis media	5 (31.2%)
	Blood system diseases: anemia, idiopathic thrombocytopenic purpura, thrombophlebitis	5 (31.2%)
	Cardiovascular diseases: hypertension, non-ischemic stress cardiomyopathy	3 (18.8%)
	Skin: psoriasis, chickenpox	3 (18.8%)
Concomitant medications (52)^a^	Steroids: prednisone, budesonide, prednisolone, methylprednisolone, beclomethasone, hydrocortisone	28 (53.8%)
	Antibacterials: amoxicillin, ceftriaxone, clavulanate potassium, cefazolin, levofloxacin, ciprofloxacin, metronidazole, fluconazole	6 (11.5%)
	ACEIs: captopril, benazepril	2 (3.8%)
	NSAIDs: indomethacin, aspirin	2 (3.8%)
	CCB: nifedipine	1 (1.9%)
	Thyroid hormones: levothyroxine	1 (1.9%)
	Hypoglycemic agent: metformin	1 (1.9%)
	Antidepressant: escitalopram	1 (1.9%)
	Antiepileptic drug: clonazepam	1 (1.9%)
	Anticoagulant: low-molecular-weight heparin	1 (1.9%)
	Other drugs for UC: azathioprine, sulfasalazine, infliximab, immunoglobulin, balsalazide	9 (17.3%)

a Represents the number of patients out of 52 in whom information regarding this particular parameter was provided.

^b^Median (minimum-maximum).

UC, ulcerative colitis; CD, Crohn’s disease; IBD, inflammatory bowel disease; ACEIs, angiotensin-converting enzyme inhibitors; CCB, calcium channel blockers; 5-ASA, 5-aminosalicylic acid; NSAIDs, Non-steroidal anti-inflammatory drugs.

### 3.2 Clinical manifestations

The clinical manifestations of the 52 patients are shown in Table 2. Forty-three (82.7%) patients had (82.7%) chest pain, 24 (46.2%) had fever, and 21 (40.4%) had respiratory symptoms. Fourteen (26.9%) patients had autonomic symptoms such as tachycardia and weakness. Thirteen (25.0%) patients had digestive symptoms such as hemafecia and belly ache. There were 21 cases (40.4%) of myocarditis, 17 cases (32.7%) of pericarditis, and 14 cases (26.9%) of myopericarditis. There were 9 cases (17.3%) with neurological symptoms, such as headache, lethargy, and syncope. Cardiovascular symptoms occurred in 6 (11.5%) patients. Eight cases (15.4%) had other clinical manifestations such as myalgia, arthralgia, and weight loss. Pericardial effusion was detected in 19 cases (36.5%).

**TABLE 2:** Clinical information of 52 included patients.

Parameter		Value
Disease type (52)^a^	Myocarditis	21 (40.4%)
	Pericarditis	17 (32.7%)
	Myopericarditis	14 (26.9%)
Clinical manifestations (52)^a^	Chest pain	43 (82.7%)
	Fever	24 (46.2%)
	Respiratory system: dyspnea, cough, flu-like symptoms, throat ache	21 (40.4%)
	Autonomic system: tachycardia, weakness, paleness, chills, sweating, fatigue	14 (26.9%)
	Digestive system: hemafecia, belly ache, nausea, vomiting, dysphagia	13 (25.0%)
	Neurological system: headache, lethargy, syncope, facial numbness	9 (17.3%)
	Cardiovascular system: hypotension, angina pectoris, heart failure, elevated blood pressure, palpitations	6 (11.5%)
	Skin: diffuse maculopapular rash, skin rash	2 (3.8%)
	Other: myalgia, arthralgia, weight loss	8 (15.4%)
Pericardial effusion (52)^a^	YES	19 (36.5%)
	NO	33 (63.5%)
Laboratory examination		
Troponin T (37)^a^	μ g/L	1.8 (0.1,165.0)^b^
	Elevated	33 (89.2%)
	Normal	4 (10.8%)
CK (24)^a^	U/L	441 (3,16000)^b^
	Elevated	16 (66.7%)
	Normal	8 (33.3%)
CRP (29)^a^	mg/L	97.1 (12.0,2580.0)^b^
	Elevated	27 (93.1%)
	Normal	2 (6.9%)
Leukocyte count (27)^a^	μ L	15,000 (7820,26,200)^b^
	Elevated	22 (81.5%)
	Normal	5 (18.5%)
ESR (20)^a^	mm/h	66.5 (16.0, 121.0)^b^
	Elevated	19 (85.0%)
	Normal	1 (5.0%)

a Represents the number of patients out of 52 in whom information regarding this particular parameter was provided.

b Median (minimum-maximum).

CK, creatine kinase; CRP, C-reactive protein; ESR, erythrocyte sedimentation rate.

### 3.3 Laboratory examination

Cardiac Troponin T (cTnT) was detected in 37 patients, including elevated levels in 33 cases (89.2%) and normal levels in 4 cases (10.8%). The median cTnT value was 1.8 μg/L (range 0.1–165.0). The serum creatine kinase (CK) assay was performed on 24 patients. Eight (33.3%) patients had normal values, and 16 (66.7%) had elevated CK levels. The median CK level was 441 U/L (range 3–16000). The C-reactive protein (CRP) test was normal in 2 cases (6.9%), and elevated in 27 cases (93.1%), totaling 29 cases. The median CRP was 97.1 mg/L (range 12.0–2580.0). Of 27 patients with leukocyte counts, 22 (81.5%) were elevated, and 5 (18.5%) were normal. The median leukocyte count was 15000/μL (range 7820–26200). The erythrocyte sedimentation rate (ESR) was determined in 20 patients. Of these, 19 cases (85.0%) were elevated, and 1 (5.0%) was normal. The median ESR was 66.5 mm/h (16.0–121.0).

### 3.4 Image examination

The summary of the imaging examination results is shown in Table 3. Twenty-three (44.2%) cases had ST-elevation on electrocardiogram (ECG), 9 (17.3%) had T wave inversion, 7 (13.5%) had sinus tachycardia, 5 (9.6%) had nonspecific ST-T wave change, 3 (5.8%) had PR interval decrease, 3 (5.8%) had biphasic T wave, 3 (5.8%) had normal ECG, 2 (3.8%) had T-wave flatness, 1 (1.9%) had an atrioventricular block, 1 (1.9%) had an increase in the Q wave, and 1 (1.9%) had trifascicular block. Eleven (91.7%) patients showed normal coronary angiography (CA), and 1 (8.3%) had atherosclerosis. Cardiac magnetic resonance imaging (CMRI) was performed in 28 cases: 12 (42.8%) had myocardial necrosis, 7 (25.0%) had myocardial edema, 2 (7.1%) had minimal pericardial effusion, 2 (7.1%) had myocardial fibrosis, 1 (3.6%) had interatrial septal hypertrophy, 1 (3.6%) had a benign pericardial cyst, 1 (3.6%) had diffuse hyperkinesia, and 1 (3.6%) had anterior septal hypertrophy. One (3.6%) patient had normal CMRI. Fifty patients had ultrasound cardiogram (UCG) performed: 15 (30.0%) had pericardial effusion, 12 (24.0%) had ventricular dysfunction, 7 (14.0%) had decreased ejection fraction, 7 (14.0%) had normal UCG, 6 (12.0%) had abnormal wall motion, and 3 (6.0%) had thickening of the ventricular wall.

**TABLE 3:** Imaging examination of 52 patients reported in case series/reports.

Parameter		Value
Electrocardiogram (52)^a^	ST elevation	23 (44.2%)
	T wave inversions	9 (17.3%)
	Sinus tachycardia	7 (13.5%)
	Non-specific ST-T wave changes	5 (9.6%)
	PR interval decrease	3 (5.8%)
	Biphasic T waves	3 (5.8%)
	Normal	3 (5.8%)
	Flattened T-waves	2 (3.8%)
	Atrioventricular block	1 (1.9%)
	Q-waves increase	1 (1.9%)
	Trifascicular block	1 (1.9%)
Coronary angiography (12)^a^	Normal	11 (91.7%)
	Atherosclerosis	1 (8.3%)
Cardiac magnetic resonance imaging (28)^a^	Myocardial necrosis	12 (42.8%)
	Myocardial edema	7 (25.0%)
	Minimal pericardial effusion	2 (7.1%)
	Myocardial fibrosis	2 (7.1%)
	Interatrial septal hypertrophy	1 (3.6%)
	Benign pericardial cyst	1 (3.6%)
	Diffuse hypokinesis	1 (3.6%)
	Anterior and septal hypertrophy	1 (3.6%)
	Normal	1 (3.6%)
Ultrasound cardiogram (50)^a^	Pericardial effusion	15 (30.0%)
	Ventricular dysfunction	12 (24.0%)
	Decreased ejection fraction	7 (14.0%)
	Normal	7 (14.0%)
	Abnormal wall motion	6 (12.0%)
	Ventricular wall thickening	3 (6.0%)

a Represents the number of patients out of 52 in whom information regarding this particular parameter was provided.

### 3.5 Treatment and prognosis

The treatment and prognosis of the 52 patients are shown in Table 4. A total of 48 patients (92.3%) eventually discontinued mesalazine, and 4 (7.7%) continued to receive mesalazine. After discontinuation of mesalazine, 25 (48.1%) patients received corticosteroids, 5 (9.6%) received azathioprine (AZA), 1 (1.9%) received balsalazide, 1 (1.9%) received infliximab (IFX), and 1 (1.9%) received cyclosporine (CsA) to treat IBD. Cardiotoxicity treatment included the use of non-steroidal anti-inflammatory drugs (NSAIDs) in 18 cases (34.6%), antibiotics in 12 cases (23.1%), surgery in 6 cases (11.5%), hypotensives in 6 cases (11.5%), analgesics in 4 cases (7.7%), vasoactive drugs in 1 case (1.9%), cardiac stimulants in 1 case (1.9%), and antianginal agents in 1 case (1.9%). Fourteen cases (33.3%) had clinical symptoms that disappeared immediately after treatment, 22 cases (52.4%) had symptoms that disappeared within a week, and 6 (14.3%) had symptoms that disappeared after more than a week. Except for one case (1.9%) whose outcome was death, all 51 cases (98.1%) recovered after treatment. Cardiotoxicity symptoms occurred in 11 cases after rechallenging with mesalazine.

**TABLE 4:** Treatment and prognosis of 52 patients reported in case series/reports.

Parameter		Value
Therapy (52)^a^	Discontinued	48 (92.3%)
	Continued	4 (7.7%)
	Treatment for IBD	25 (48.1%)
	Corticosteroids	5 (9.6%)
	Azathioprine	1 (1.9%)
	Balsalazide	1 (1.9%)
	Infliximab	1 (1.9%)
	Cyclosporine	
	Treatment for cardiotoxicity	18 (34.6%)
	NSAIDs	12 (23.1%)
	Antibiotics	6 (11.5%)
	Surgery: subtotal pericardectomy, pericardiocentesis, cardiac pacemaker implantation	6 (11.5%)
	Hypotensive drugs	4 (7.7%)
	Analgesics	1 (1.9%)
	Vasoactive drugs	1 (1.9%)
	Cardiac stimulants	1 (1.9%)
	Antianginal agents	
Symptom disappearance time (42)^a^	Immediately	14 (33.3%)
	0-7d	22 (52.4%)
	>7d	6 (14.3%)
Time of cardiotoxicity after mesalazine re-challenge (11)^a^	Immediately	3 (27.3%)
	0-7d	7 (63.6%)
	>7d	1 (9.1%)
Prognosis (52)^a^	Recover	51 (98.1%)
	Death	1 (1.9%)

a Represents the number of patients out of 52 in whom information regarding this particular parameter was provided.

IBD, inflammatory bowel disease; NSAIDs, Non-steroidal anti-inflammatory drugs.

## 4 Discussion

The FDA first approved mesalazine in 1992 to treat IBD (Lahiff et al., 2011). Its dosage forms include tablets, suppositories, capsules, and granules. The recommended dose for oral administration of mesalazine is 2 g/day, and the recommended dose for rectal administration is 3 g/week in divided doses (Harbord et al., 2017). Mesalazine inhibits prostaglandin formation by inhibiting cyclooxygenase (COX). It reduces signaling through the peroxisome proliferator-activated receptor gamma (PPAR-γ) pathway, decreasing nuclear factor ĸB activity and colon inflammation (Kim et al., 2006). Most of the cases in this study took the recommended dose, and only three patients received a higher amount. Therefore, cardiotoxicity due to mesalazine may not be correlated with the dose.

The onset time of cardiotoxicity for most patients was 2–4 weeks after taking mesalazine, suggesting that we need to pay attention to patients who develop fever, chest pain, and breathing difficulties, especially in the early stages (Radhakrishnan et al., 2018; Shergill, 2021). However, in some patients, the onset of cardiotoxicity was delayed for several months to several years (Okoro et al., 2018; Caio et al., 2021). It is important to note that the onset time does not shorten after increasing the dose of mesalazine, which may indicate that the onset time is not correlated with the dose (Kaiser et al., 1997; Perez-Colon et al., 2011). Almost 80% of patients with heart injury were men, suggesting that sex may be an independent risk factor for mesalazine-caused myocarditis. Most patients did not have cardiovascular diseases such as hypertension and nonischemic cardiomyopathy. Therefore, the cardiovascular disease may not be an independent risk factor for mesalazine cardiotoxicity. Ciprofloxacin, levofloxacin, and infliximab have been reported to be cardiotoxic (Lezcano-Gort et al., 2015; Dipasquale et al., 2018; Meng et al., 2019). Concomitant use of other cardiotoxic agents may be a risk factor for myocarditis caused by mesalazine.

Identifying the primary cause of mesalazine-induced cardiotoxicity is challenging because cardiotoxicity is a rare manifestation of IBD (Sorensen and Fonager, 1997; Oh et al., 2012). Cardiotoxicity from IBD typically manifests itself as pericarditis, myocarditis, myocardial infarction, and heart failure (Ibrahim et al., 2019; Rogler et al., 2021). Cardiac adverse reactions in patients with IBD treated with mesalazine are rare. The specific mechanisms by which mesalazine causes heart damage are unclear. Several possible mechanisms have been proposed. Mesalazine inhibits COX1 and accelerates the arachidonic acid metabolism into lipoxygenase products. Excess lipooxygenase products induce pro-inflammatory signaling, causing allergic myocarditis by releasing eosinophil-stimulating cytokines (Merceron et al., 2010). Mesalazine-induced pericarditis may be a humoral-mediated hypersensitivity response in which antibodies produced against mesalazine cross-react with heart tissues, leading to inflammation (Sentongo and Piccoli, 1998; Waite and Malinowski, 2002). Other possible mechanisms include the direct cardiotoxic effects of mesalazine and lgE- or cell-mediated hypersensitivity (Kaiser et al., 1997). In addition, mesalazine induces reactive oxygen species formation and the mitochondrial membrane potential collapse in rat cardiac mitochondria. This causes mitochondrial dysfunction and cytochrome c release, eventually leading to cardiomyocyte apoptosis and cardiovascular dysfunction (Salimi et al., 2020).

Clinical manifestations of mesalazine-induced cardiotoxicity are diverse and nonspecific, including fever, chest pain, and dyspnea. cTnT and CK are specific markers of myocardial injury (Janardhanan, 2016). Most patients with cardiotoxicity had significantly increased biochemical markers such as cTnT, CK, and CRP. Leukocyte count and ESR also increased significantly. Once clinicians suspect a patient has mesalazine-induced cardiotoxicity, they should confirm the diagnosis by promptly examining the cardiac biochemical markers and initiating symptomatic treatment.

Imaging is an essential method for evaluating drug-induced cardiotoxicity. It can effectively assess structural and functional changes secondary to myocarditis or pericarditis. Imaging includes ECG, echocardiography, and CMRI (Nieminen et al., 1984; Valbuena-Lopez et al., 2016). Patients with cardiotoxicity usually show nonspecific ST-segment elevation or T-wave inversion on the ECG. Echocardiography can identify pericardial effusion or left cardiac insufficiency. CMRI can assess myocardial necrosis or myocardial edema. Cardiac imaging is recommended at the beginning of mesalazine treatment and follow-ups for early prevention or intervention of possible cardiotoxicity.

Treatment for cardiotoxicity is the immediate discontinuation of mesalazine. Most patients usually have symptoms that disappear within a few days. The toxic effects of the drug can lead to the development of immune-mediated acute myocarditis. The first-line treatment option for such immune-mediated acute myocarditis is discontinuing the medication and starting corticosteroids (Ammirati et al., 2020). Alemtuzumab and anti-thymocyte globulin are available as second-line treatments (Jain et al., 2018; Esfahani et al., 2019). The benefits of corticosteroids have not been compared in patients who received the therapy with those who did not (Brown, 2016). Patients usually wish to resume IBD treatment with mesalazine. However, rechallenging can lead to cardiotoxicity symptoms reappearing within hours or days (Agnholt et al., 1989; Gujral et al., 1996; Coman et al., 2014). Therefore, mesalazine rechallenge or switching to an alternative drug that does not contain 5-ASA should be carefully considered.

The study has the following limitations. First, the quality of the included studies was poor. More high-quality prospective cohort studies are needed. Second, only electronic databases were searched, and full texts of some studies could not be obtained, which can lead to selection and information bias.

## 5 Conclusion

Cardiotoxicity is a rare and serious adverse effect of mesalazine. Clinicians should consider the possibility of cardiotoxicity in patients with fever, chest pain, dyspnea, and other symptoms, especially within 4 weeks of treatment. Immediate discontinuation of mesalazine is necessary. Corticosteroids can improve patient symptoms, leading to a good prognosis. Laboratory tests (cTnT, serum CK) and imaging (ECG, echocardiogram, and cardiac MRI) should be performed.

## 6 Future prospects

As the number of patients diagnosed with IBD increases worldwide, more patients are exposed to mesalamine. Further rigorous experiments are needed to clarify the specific mechanism of mesalamine-induced cardiotoxicity. Multicenter prospective cohort studies with more rigorous designs, larger sample sizes, and higher qualities are necessary to identify high-risk groups and explore optimal treatment options. In patients who developed cardiotoxicity, follow-ups should be provided to observe the long-term prognosis of patients.

## Data Availability

The original contributions presented in the study are included in the article/supplementary material, further inquiries can be directed to the corresponding authors.
